# Exome sequencing in one family with gastric- and rectal cancer

**DOI:** 10.1186/s12863-016-0351-z

**Published:** 2016-02-13

**Authors:** Jessada Thutkawkorapin, Simone Picelli, Vinaykumar Kontham, Tao Liu, Daniel Nilsson, Annika Lindblom

**Affiliations:** Karolinska Institutet, Department of Molecular Medicine and Surgery, Stockholm, Sweden; Eukaryotic Single Cell Genomics facility, Science for Life Laboratory, Stockholm, Sweden

**Keywords:** Next Generation Sequencing, Exome sequencing, Bioinformatics, Familial, Colorectal cancer, Genetics

## Abstract

**Background:**

Heritable factors are well known to increase the risk of cancer in families. Known susceptibility genes account for a small proportion of all colorectal cancer cases. The aim of this study was to identify the genetic background in a family suggested to segregate a dominant cancer syndrome with a high risk of rectal- and gastric cancer. We performed whole exome sequencing in three family members, 2 with rectal cancer and 1 with gastric cancer and followed it up in additional family members, other patients and controls.

**Results:**

We identified 12 novel non-synonymous single nucleotide variants, which were shared among 5 affected members of this family. The mutations were found in 12 different genes; *DZIP1L*, *PCOLCE2*, *IGSF10*, *SUCNR1*, *OR13C8*, *EPB41L4B*, *SEC16A*, *NOTCH1*, *TAS2R7*, *SF3A1*, *GAL3ST1*, and *TRIOBP*. None of the mutations was suggested as a high penetrant mutation. It was not possible to completely rule out any of the mutations as contributing to disease, although seven were more unlikely than the others. Neither did we rule out the effect of all thousands of intronic, intergenic and synonymous variants shared between the three persons used for exome sequencing.

**Conclusions:**

We propose this family, suggested to segregate dominant disease, could be an example of complex inheritance.

## Background

Colorectal cancer (CRC) is the second most common cancer type in Sweden and the third most common cancer type in the western world. Epidemiological studies have estimated that the risk of developing colorectal cancer in first-degree relatives of patients diagnosed with cancer is increased by two to four-fold [1]. Several hereditary syndromes, such as Familial Adenomatous Polyposis (FAP) and Lynch syndrome, are known where the risk of cancer development can be as high as 100 %. However, all known familial CRC syndrome account for less than 5 % of all colorectal cancer cases. No hereditary cause has been identified in most of the families with familial cancer. Even though these families show empirical evidence of an increased risk of developing cancer, most of them do not fulfill the criteria for FAP or Lynch syndrome [2]. This is indicative of additional genes predisposing to cancer development, which are yet to be discovered. Linkage studies in familial CRC have been successful in localizing highly penetrant CRC genes such as *APC*, *MSH2*, *MLH1,* and recently also *GREM1* [3, 4]. More recent studies using linkage analysis in familial CRC have resulted in various mostly non-overlapping suggested loci. Only one locus on chromosome 9 has been confirmed in several studies [5–7]. Other studies have focused on studying CRC as a complex disease and presented evidence for low penetrant genetic risk factors, each typically with a very small increased risk of cancer. Till date, 25 variants have been suggested [8]. Next generation sequencing (NGS) has become a valuable tool in the discovery of candidate genes in several studies. So far, this has generated only a small number of potential CRC predisposing genes such as *POLE, POLD1,* and *NTHL1* [9, 10]. The likelihood of identifying high-penetrant genes is increased by using large pedigrees with familial cancer as exemplified by the findings of *FAN1* [11]. The combination of linkage analysis and NGS of the target region using large pedigrees has also been successful to define *BMPR1A* and *RPS20* as predisposing genes [12, 13].

We have previously published a linkage study reporting a LOD score of 2.1 in a region on chromosome 3q [14]. One large pedigree (family 242) mostly contributed to this high LOD score, where a seemingly dominant predisposition to rectal and/or gastric cancer was observed. We hypothesized that the mixed representation of rectal and gastric cancer among family members was due to one predisposing mutation in one gene and performed a whole exome study to test it. Three family members were chosen for whole-exome sequencing; one case with gastric cancer at age 63, and two cases with rectal cancer at age 50 and 40 years of age respectively.

## Methods

### Family 242

The family segregates early onset rectal- and gastric cancer over three generations suggesting a dominant inherited predisposition. In total there were six cases with early-onset rectal cancer and in total at least four cases with gastric cancer. Many family members had presented with tubular adenomas and hyperplastic polyps under surveillance. In particular, four family members had lesions, which could be used for coding of affected status in our study. One (Co-652) had three large tubulovillous adenomas (TVA), one (Co-692) had four tubular adenomas (TA) and 8 hyperplastic polyps (HP), and one (Co-657) had 5 large HP. They were all coded as affected in the first linkage analysis. One family member with gastric cancer (Co-441) and two relatives with rectal cancer (Co-666 and Co-771) were used for the initial exome sequencing study (Table 1).

**Table 1:** Segregation test of 34 variants in family 242

Chrom:Position^a^	Ref	Alt	Gene	dbSNP	1000G^b^	Co-441	Co-634	Co-666	Co-667	Co-771	Co-652	Co-692	Co-657
1:235577776	C	T	TBCE	rs62620041	0.0023	C/T	C/C	C/T	C/T	C/T	C/C	C/C	C/C
2:29295186	C	T	C2orf71	rs75276619	0.01	C/T	C/T	C/T	C/C	C/C	C/C	C/T	C/C
2:155555406	A	G	KCNJ3	rs16838016	0.0032	A/G	A/A	A/G	A/G	A/G	A/G	A/A	A/G
2:169870004	G	A	ABCB11	rs11568361		A/G	G/G	A/G	A/G	A/G	A/G	G/G	G/G
3:137786409	A	C	DZIP1L			A/C	A/C	A/C	A/C	A/C	A/C	A/A	A/C
3:142542415	C	T	PCOLCE2	rs147612568	0.0014	C/T	C/T	C/T	C/T	C/T	C/T	C/C	C/T
3:151171329	G	T	IGSF10	rs143721392		G/T	G/T	G/T	G/T	G/T	G/T	G/T	G/T
3:151598890	T	C	SUCNR1			C/T	C/T	C/T	C/T	C/T	C/T	C/T	C/T
4:13590380	A	G	BOD1L1	rs140964488	0.0009	A/G	A/G	A/G	A/A	A/G	A/A	A/G	A/A
4:22440018	C	G	GPR125	rs144997202	0.0005	C/G	C/G	C/G	C/C	C/G	C/G	C/G	C/C
4:25849449	G	A	SEL1L3			A/G	A/G	A/G	G/G	A/G	A/G	A/G	G/G
7:141765172	A	T	MGAM			A/T	A/T	A/T	A/A	A/T	A/A	A/A	A/A
9:107331452	G	A	OR13C8			A/G	A/G	A/G	A/G	A/G	G/G	G/G	G/G
9:111947836	GGA	-	EPB41L4B			GGA/-	GGA/-	GGA/-	GGA/-	GGA/-	GGA/GGA	GGA/GGA	GGA/GGA
9:139369066	G	A	SEC16A	rs148167113	0.01	A/G	A/G	A/G	A/G	A/G	A/G	A/G	A/G
9:139401233	C	T	NOTCH1	rs61751543	0.01	C/T	C/T	C/T	C/T	C/T	C/T	C/T	C/T
10:5931230	C	T	ANKRD16	rs3750659	0.06	C/T	C/T	C/T	C/C	C/T	C/C	C/C	C/C
11:130784886	T	C	SNX19	rs117260465	0.01	C/T	C/T	C/T	T/T	C/T	T/T	C/T	T/T
12:10954583	A	T	TAS2R7	rs139604652	0.0032	A/T	A/T	A/T	A/T	A/T	A/A	A/T	A/A
12:109617728	A	G	ACACB	rs16940029	0.08	A/G	A/A	A/G	A/A	A/G	A/A	A/G	A/G
16:14029033	G	A	ERCC4	rs1800067	0.03	A/G	G/G	A/G	A/G	A/G	G/G	G/G	A/G
16:15818842	A	G	MYH11	rs16967510	0.02	A/G	A/A	A/G	A/A	A/G	A/G	A/A	A/G
16:22826046	T	G	HS3ST2	rs189013090	0.01	G/T	T/T	G/T	G/T	G/T	T/T	T/T	G/T
17:62028920	C	G	SCN4A	rs41280102	0.01	C/G	C/C	C/G	C/C	C/G	G/G	C/G	C/G
18:67721492	G	C	RTTN	rs34717557	0.01	C/G	G/G	C/G	G/G	C/G	C/G	G/G	C/G
18:67836115	G	T	RTTN	rs34353615	0.01	G/T	G/G	G/T	G/G	G/T	G/T	G/G	G/T
18:72343156	A	G	ZNF407	rs75994611	0.01	A/G	A/A	A/G	A/A	A/G	A/G	A/A	A/G
19:3834863	C	T	ZFR2	rs61747120	0.04	C/T	C/C	C/T	C/C	C/T	C/T	C/T	C/T
22:30733787	C	T	SF3A1			C/T	C/T	C/T	C/T	C/T	C/T	C/T	C/C
22:30951208	C	T	GAL3ST1	rs139452633	0.0005	C/T	C/T	C/T	C/T	C/T	C/T	C/T	C/C
22:38111897	C	T	TRIOBP	rs143157673	0.0018	C/T	C/T	C/T	C/T	C/T	C/T	C/T	C/C
22:46653273	C	T	PKDREJ	rs147180698		C/T	C/C	C/T	C/C	C/T	C/T	C/T	C/C
X:107844666	G	T	COL4A5	rs34077552	0.01	G/T	G/G	G/T	G/T	T/T	G/T	G/T	G/T
X:119293216	-	G	RHOXF2			-/G	−/−	-/G	-/G	-/G	-/G	-/G	-/G

List of 34 rare variants after filtering all non-exonic variants, synonymous variants, variants presenting in the 30 breast cancer cases [15], and variants with allele frequency in 1000Genomes more than 20 %

^a^GRCh37 (hg19) coordinates

^b^1000 Genomes version April 2012 (hg19)

### Samples used in analysis

Exome sequencing of three members from family 242 was performed along with 30 research samples sequenced for a different study [15]. The data was used in addition to MAF to rule out common variants, as these samples used the same library preparation, same sequencing facility at the same time. No information was recorded from any individual patients in this study.

Anonymous exome data from 249 consenting rare disease patients and relatives from the department of Clinical Genetics at Karolinska University Hospital, Solna, Sweden (249 Swedish controls) were used for comparison of allele frequencies in our analysis. An additional dataset of 98 cases from 57 high-risk colorectal cancer families, who had undergone whole-exome sequencing (unpublished data), was also used for comparison. The families were included for study when they underwent genetic counseling at the department of Clinical Genetics, Karolinska University Hospital, Solna (Sweden). Finally, in total 190 cases from 190 families with at least two gastric and one colorectal, or at least two colorectal and one gastric cancer cases were used for testing of the candidate gene *SUCNR1*. The families were included in studies as part of the Swedish Colorectal Cancer Low-risk Study, which included consecutive CRC cases between 2003 and 2009.

The study was undertaken with permission for the ¨Regional research ethics committee in Stockholm, ID´s: 2002/489 (Swedish Colorectal Cancer Low-risk Study) and 2008/125-31.2 (participants recruited from dept of Clinical Genetics) and 2012/2106-31.4 (The 249 Swedish controls). All participants gave written consent to participate in the studies.

### Exome sequencing family 242 and 30 other research samples

Library preparation was performed with the SureSelect XT Human All Exon 50 Mb kit. Samples were clustered on a cBot and sequenced on Illumina HiSeq 2000. The reads were aligned to the reference genome hg19GRCh37 using BWA [16]. Then, the calculation of mapping and enrichment statistics were done with Picard [17] and GATK [18]. The average coverage of samples Co-441, Co-666, and Co-771, are 41x, 32x, and 35x. And the percent of bases above 15x are 80.2 %, 72.9 %, and 76.3 % respectively.

### Exome sequencing of 98 familial CRC samples

DNA was quantified using a Qubit Flurometer (Life Technologies). Sequencing libraries were prepared according to the TruSeq DNA Sample Preparation Kit EUC 15005180 or EUC 15026489 (Illumina). Briefly, 1–1.5 ug of genomic DNA was fragmented using a Covaris (Covaris, Inc.). Thirty-seven of the DNA samples were fragmented according to the Covaris 400 bp protocol and 61 samples were fragmented according to the SureSelect Protocol. After fragmentation, all samples were subjected to end-repair, A-tailing, and adaptor ligation of Illumina Multiplexing PE adaptors. An additional gel-based size selection step was performed for the 37 samples. The adapter-ligated fragments were subsequently enriched by PCR followed by purification using Agencourt AMPure Beads (Beckman Coulter). Exome capture was performed by pre-pooling equimolar amounts and performing enrichment in 5- or 6-plex reactions according to the TruSeq Exome Enrichment Kit Protocol (EUC 15013230). Library size was checked on a Bioanalyzer High Sensitivity DNA chip (Agilent Technologies) while concentration was calculated by quantitative PCR. The pooled DNA libraries were clustered on a cBot instrument (Illumina) using the TruSeq PE Cluster Kit v3. Paired-end sequencing was performed for 100 cycles using a HiSeq 2000 instrument (Illumina) with TruSeq SBS Chemistry v3, according to the manufacturer’s protocol. Base calling was performed with RTA (1.12.4.2 or 1.13.48) and the resulting BCL files were filtered, de-multiplexed, and converted to FASTQ format using CASAVA 1.7 or 1.8 (Illumina). Data have been analyzed using the bcbb package [19]. After sequencing, the samples have been aligned to the reference genome hg19GRCh37 using BWA, sorted and PCR duplicates were removed with Picard. The calculation of mapping and enrichment statistics were done with Picard and GATK. Variants were called using GATK and followed a best practice procedure implemented at the Broad Institute [20].

### Sanger sequencing

The PCR primers were designed using Primer3web [21] and SimGene Primer3 [22]. The sequences were visualized and analyzed using FinchTV [23] and CodonCode Aligner [24].

### Mutation annotation

The output mutations in variant call format (vcf) were annotated using ANNOVAR [25], which generated an Excel-compatible file with gene annotation, amino acid change annotation, dbSNP identifiers [26], 1000 Genomes Project allele frequencies [27], and functional prediction from SIFT [28], PolyPhen2 [29], LRT [30], MutationTaster [31], PhyloP [32], and GERP++ [33].

## Results

Whole exome-sequencing was used to analyze the three patients from family 242 together with 30 other research samples for a separate study. All samples were computationally analyzed using a process to generate candidate mutations to be causative in family 242. All mutations shared between the three family members were selected, all with a MAF > 20 % in 1000Genomes (1000G), all non-exonic and synonymous variants, and all variants present in more than one of the 30 other research samples were excluded. After this filtering 34 mutations/variants remained as candidates (Table 1). Interestingly, not only the region on chromosome three showed linkage to cancer in the family but also several other chromosomal regions (Table 1). We used another five relatives from this kinship for Sanger sequencing of the 34 variants to find out the correlation with disease. The outcome for each family member is shown in Table 1.

Of the five family members tested for the 34 variants, only two (Co-634 and Co-667) had cancer, and both had rectal cancer and were therefore considered to be gene carriers. Using this data allowed us to remove 22 of the 34 variants. In detail, 15 variants were excluded, since they were not shared by Co-634. Seven more were excluded since they were not shared by Co-667 (Tables 1 and 2). Thus, 12 candidate mutations in four chromosomal regions remained as predisposing gene mutation candidates. All twelve variants were either unique (not present in 1000G) or extremely rare (1000G MAF < 1 %). The *EPB41L4B* has an in-frame deletion of three bases in exon 23, and all other mutations were missense mutations. Five of them had already been reported in dbSNP. The mutation frequency of these 12 mutations was compared to 98 Swedish familial CRC cases, 249 Swedish controls, and MAF in 1000G. Only three of the 12 variants were present among 98 familial CRC cases (in the genes *SEC16A*, *NOTCH1* and *TAS2R7*) (Table 2). However, none of those three segregated with the disease in the other families, and thus, cannot be regarded as high-risk gene-mutations.

**Table 2:** Twelve candidate mutations in family 242, and in-silico functional prediction

Gene	dbSNP	1000G	98 CRC cases	249 patients	F1	F2	F3	F4	F5
DZIP1L					C	T	D	N	N
PCOLCE2	rs147612568	0.0014			C	D	D	D	D
IGSF10	rs143721392				C	D	D	D	N
SUCNR1					C	D	D	D	N
OR13C8					C	T	B	N	-
EPB41L4B				0.002	Non-frameshift deletion				
SEC16A	rs148167113	0.01	0.0153	0.004	-	-	-	-	-
NOTCH1	rs61751543	0.01	0.0204	0.01	C	T	D	D	D
TAS2R7	rs139604652	0.0032	0.0051		C	D	D	N	N
SF3A1					C	T	P	D	D
GAL3ST1	rs139452633	0.0005		0.004	C	D	D	D	D
TRIOBP	rs143157673	0.0018			N	-	P	-	-

F1, Phylop; C, Conserved; N, Not conserved

F2, SIFT; T, Tolerated; D, Deleterious

F3, Polyphen2; D, Probably damaging; P, Possibly damaging; B, Benign

F4, LRT; N, Neutral; D, Deleterious

F5, MutationTaster; N, Polymorphism; D, Disease causing

Next, we used our 98 CRC cases to search for other mutations in the 12 genes. We excluded all non-exonic and synonymous variants, all variants with MAF > 20 %, and those without any predicted pathogenic effect, and variants with a frequency less than the Swedish controls. After this, 36 variants among 11 genes remained (Table 3). No additional mutation was seen in *SUCNR1*. To find out if *SUCNR1* could represent a high-penetrant gene, 190 samples from families with both colorectal and gastric cancer were used for sequencing of the whole gene without finding any mutation. The *SUCNR1* functions as a receptor for the citric acid cycle intermediate succinate, involved in the renin-angiotensin system [34] and from its function less likely to be associated with a colorectal cancer risk. Thus, we could not find any further support for *SUCNR1* as a candidate gene. One interesting candidate variant was a frameshift deletion in the *TRIOBP* gene but it did not segregate in a family. Another variant was a non-frameshift deletion in the *SEC16A* gene but it did not segregate with cancer in the family either. One other potential mutation was a stop-gain in the *DZIP1L* gene but it also did not segregate in the family. All other 33 mutations were non-synonymous SNPs. Analysis in other families showed segregation only in one family, where a variant in the gene *IGSF10* was shared between two affected relatives. However, the same variant was also found in three other families where it did not segregate with disease. Thus, none of the 12 genes was supported as being a high-penetrant gene variant based on the analysis of the 98 families colorectal cancer cases (Table 3).

**Table 3:** Thirty-six mutations in the twelve genes that can be found in 98 CRC cases

Chrom:Position^a^	Ref	Obs	dbSNP	Func	Gene	Exonic function	1000G	249 patients	98 CRC cases^b^	F1	F2	F3	F4	F5	^c^	^d^	^e^
3:137786496	G	A		exonic	DZIP1L	stopgain SNV			0.0051	N	-	-	N	D			1
3:137790616	C	T	rs150466957	exonic	DZIP1L	ns SNV	0.0009		0.0051	N	D	P	N	N			1
3:137813726	G	A	rs148594666	exonic	DZIP1L	ns SNV	0.0009		0.0051	C	T	P	N	N	1		
3:142539852	C	T		exonic	PCOLCE2	ns SNV		0.002	0.0051	C	T	B	D	N			1
3:142548681	C	T	rs140721173	exonic	PCOLCE2	ns SNV	0.0009	0.002	0.0051	N	T	B	D	D			1
3:151163838	T	C	rs34114908	exonic	IGSF10	ns SNV	0.01	0.01	0.0357	N	D	B	N	N		2	3
3:151165241	G	C	rs142202060	exonic	IGSF10	ns SNV	0.0005	0.004	0.0102	C	D	P	N	N		1	
3:151165532	G	C	rs35667704	exonic	IGSF10	ns SNV	0.0037	0.0141	0.0153	N	D	P	N	N		1	1
3:151166124	A	G	rs146828199	exonic	IGSF10	ns SNV			0.0051	C	D	D	N	D			1
9:107332377	T	A	rs76017116	exonic	OR13C8	ns SNV	0.01	0.004	0.0153	C	D	B	D	D			3
9:111954616	C	T	rs199718023	exonic	EPB41L4B	ns SNV			0.0051	C	D	B	N	N			1
9:111954622	C	T	rs201059767	exonic	EPB41L4B	ns SNV			0	C	D	B	N	N			1
9:112029768	C	T		exonic	EPB41L4B	ns SNV			0.0051	C	T	B	N	-	1		
9:112082510	C	T	rs117569740	exonic	EPB41L4B	ns SNV	0.14	0.1124	0.1531	C	D	B	-	-	8	4	14
9:139345847	G	A	rs45519739	exonic	SEC16A	ns SNV	0.01		0.0153						1		1
9:139348749	G	A	rs79974534	exonic	SEC16A	ns SNV	0.01	0.0141	0.0306						1	1	2
9:139360781	G	A	rs199798606	exonic	SEC16A	ns SNV		0.004	0.0051								1
9:139368953	G	A	rs3812594	exonic	SEC16A	ns SNV	0.16	0.2048	0.2653						9	9	17
9:139369066	G	A	rs148167113	exonic	SEC16A	ns SNV	0.01	0.004	0.0153						1		2
9:139369091	C	T	rs11788702	exonic	SEC16A	ns SNV	0.0005		0.0051								1
9:139369408	C	T	rs200238338	exonic	SEC16A	ns SNV			0.0051						1		
9:139369816	G	C	rs200394508	exonic	SEC16A	ns SNV			0.0204						1		3
9:139370955	del9	-		exonic	SEC16A	non-frameshift deletion		0.008	0.0153						1		2
9:139401233	C	T	rs61751543	exonic	NOTCH1	ns SNV	0.01	0.01	0.0204	C	T	D	D	D	2		2
9:139409775	C	T	rs201077220	exonic	NOTCH1	ns SNV	0.0009		0.0051	C	T	B	-	D			1
12:10954258	C	T	rs619381	exonic	TAS2R7	ns SNV	0.07	0.0884	0.1173	N	T	D	N	N	1	5	12
12:10954583	A	T	rs139604652	exonic	TAS2R7	ns SNV	0.0032		0.0051	C	D	D	N	N			1
22:30742345	T	G		exonic	SF3A1	ns SNV			0.0102	C	T	P	D	D			2
22:30953280	C	T	rs55674628	exonic	GAL3ST1	ns SNV	0.01	0.0161	0.0204	C	T	B	N	N	2		3
22:38120338	G	A		exonic	TRIOBP	ns SNV			0.0051	N	-	D	-	-	1		
22:38120542	C	T	rs142024473	exonic	TRIOBP	ns SNV	0.03	0.0201	0.0459	C	-	P	-	-	1	1	6
22:38120985	A	G		exonic	TRIOBP	ns SNV			0.0051	N	-	D	-	-	1		
22:38121040	C	T	rs41296243	exonic	TRIOBP	ns SNV	0.01	0.0201	0.0306	N	-	P	-	-	2	1	2
22:38121795	C	T	rs200359708	exonic	TRIOBP	ns SNV	0.0018		0.0051	C	-	D	-	-	1		
22:38122414	AG	-		exonic	TRIOBP	frameshift deletion			0.0051								2
22:38129388	G	A	rs34066624	exonic	TRIOBP	ns SNV	0.0023	0.008	0.0204	C	-	-	-	-	1		3

^a^GRCh37 (hg19) coordinates

^b^1000 Genomes version April 2012 (hg19)

^c^Number of families where only one individual was sequenced and had a mutation

^d^Number of families where at least two individuals were sequenced and the mutation segregated

^e^Number of families where at least two individuals were sequenced and the mutation did not segregate

F1, Phylop; C, Conserved; N, Not conserved

F2, SIFT; T, Tolerated; D, Deleterious

F3, Polyphen2; D, Probably damaging; P, Possibly damaging; B, Benign

F4, LRT; N, Neutral; D, Deleterious

F5, MutationTaster; N, Polymorphism; D, Disease causing

We considered the known functions of the genes to predict if they were likely CRC genes. We also considered the predicted pathogenicity of each conceptual non-synonymous amino acid change. All 12 variants but one (*SEC16A*) were predicted to have a pathogenic effect based on at least one predictor algorithm (Table 2). The *SUCNR1* we already excluded as a candidate high-risk mutation (above). The genes *PCOLCE2*, *SEC16A*, *TAS2R7*, and *TRIOBP* were considered less likely to be associated with increased CRC risk based on established functions. The Pro-collagen C-endopeptidase enhancer (*PCOLCE2*)*,* has no known relation to cancer [35]*.* The S. Cerevisiae homolog (*SEC16A*), is a peripheral membrane protein and is required for protein transport from ER to Golgi [36]. The Taste receptor (*TAS2R7*) is a member of the G protein–coupled receptor superfamily and specifically expressed in taste receptor cells [37]. Trio- and F-actin-binding protein (*TRIOPB*) has been related to autosomal recessive deafness syndromes [38].

The linkage study performed previously used also those with advanced polyps as affected in analysis [14]. We tested also in this study to use polyps in relatives to select among the genes. The patient (Co-652) with three tubulovillous adenomas (all in rectum and two with high-degree dysplasia) at the first colonoscopy, was highly likely to be a gene carrier. Making this assumption, two more genes (*OR13C8, EPB41L4B*) could be excluded. The patient (Co-692) with four small tubular adenomas at an age of 75 was a less clear case. The adenomas were 2 mm each and located in the ascending, transverse and descending colon, all with low-degree dysplasia, with an additional one in rectum with high-degree dysplasia. To consider this individual as affected and a gene carrier would exclude one more candidate gene (*DZIP1L*). Finally, if also the patient (Co-657) with five hyperplastic polyps at an age of 73 years was considered a gene carrier, yet another three genes (*SF3A1, GAL3ST1, TRIOBP*) could be excluded.

## Discussion

Many pedigrees in families seeking counseling about their risk of cancer show a pedigree of typical dominant high-penetrant disease. Family 242 seemed to segregate a risk of rectal as well as gastric cancer and perhaps other cancers among the family members. The pedigree suggested a mutation in a highly penetrant predisposing gene. When the family was tested negative for known inherited syndromes it was included in studies to localize new disease genes. First, linkage analysis was employed assuming a dominant mode of inheritance and this resulted in a candidate region on chromosome 3 [14]. The region was quite large and it was not possible at the time to perform sequencing of all genes in the region. Only a limited number of candidate genes were studied without finding a clear mutation [14]. When massively parallel sequencing (MPS) became feasible we decided to study the family further and performed exome sequencing for three family members. First, all genes in the region on chromosome 3 was studied, without finding any clear candidate gene. Next, the whole exome was studied. It was clear that the three studied family members shared several chromosomal regions (Table 1) and not just the one we had detected in our linkage study. When studying the linkage data again we could see that linkage was not excluded but did not generate a high enough LOD score to be considered candidates. We could identify up to five or even 12 different genes and mutations, which all could have contributed more or less to the development of tumors in this family. There was no evidence to directly pinpoint one of them, and there was at the same time some evidence to support the conclusion that none of the mutation would be associated with a high risk, and being high penetrant.

Several explanations for our findings are possible. First, some issues could be related to failures in interpretation of MPS data. How the sequences are aligned depends on the algorithm used. Different algorithms or parameters used at different sequencing centers may result in different alignments and different variants that are called, especially in the case of insertions or deletions. Old sequence processing workflow may not be able to detect large deletions (more than 10 bps) in a correct way. It is possible that a deletion could have been interpreted as several different point mutations. We could also have missed a mutation by exclusion of intronic, intergenic and synonymous mutations. However, it would have been very difficult to functionally prove the association of such variants with the disease.

Second, we could have used the wrong individuals for our first experiment. In the case one of the three is actually a phenocopy, or if there are two traits, one with high-penetrant gastric cancer and one with high-penetrant rectal cancer, it would have been missed in the analysis. Considering all patients with gastric or rectal cancer as affected is quite safe and in particular when the age of onset is low (which was the case for all rectal cancers). The use of advanced adenomas at an early age is also frequently used in studies as substitute for colorectal cancer. The ages of onset of gastric cancer in our study were 63, 63, 72 and 74 respectively, why it was reasonable to assume our case of gastric cancer first sequenced (aged 63) as affected in our hypothesis of one gene – two diseases. It is possible that instead of one high-penetrant gene, there is a polygenic mode of inheritance where more than one mutation could have contributed to the development of both gastric and rectal cancer. It is also possible that there are two different low-penetrant genes for gastric and rectal but with same or different modifying gene mutations among family members.

Previous linkage studies have identified several candidate regions on different chromosomes, but the only one in the present study, which resembles any of the published regions, is 9q [6, 7, 14, 39–41]. The region identified in the present study (the variants in the genes *OR13C8, EPB41L4B, SEC16A* and *NOTCH1*), is just proximal to that region on 9q. It is possible that the published locus and the one in the present study are really the same and that it holds a modifier gene acting only with the rectal cancers and not on the gastric cancer. If this is the case the gene of interest here would be the *NOTCH1* or another gene within the same locus.

Of the 12 candidate variants found in the family 242, seven were less likely due to our analysis above. This means that there are at least five genes as candidates to have contributed to the disease in the family (*DZIP1L, IGSF10, NOTCH1, SF3A1, GAL3ST1*). The *NOTCH1* gene is well known to be involved in cancer. The gene has been suggested to be involved specifically in both colorectal and gastric cancer, although, so far it has not been found to confer an increased risk [42, 43]. The *NOTCH1* variant in our family was found in three other families, where it did not segregate. This does not exclude an effect, but does not suggest it to be high penetrant. The other four candidate mutations showed to be mutated in several of the 98 familial colorectal cancer cases. However, none was suggested to be a high-penetrant mutation based on segregation analysis in this dataset described above. The human Iguana gene *DZIP1L* has been suggested to be part of the Hedgehog signaling pathway, which is often activated in gastric cancer but not often in colorectal cancer [44, 45]. The *GAL3ST-2* has been shown to be involved in CRC and gastric cancer [46, 47] while *GAL3ST-1* has only been suggested to be involved in ovarian cancer [48]. The *IGSF10* gene has not been described in relation to colorectal or gastric cancer but is a gene involved in differentiation and developmental processes, and possibly involved in rat osteosarcomas [49]. The gene *SF3A1* was studied in relation to CRC adenomas without finding any correlation to this gene [50].

## Conclusion

We did not find any clear high-risk gene mutation to explain the seemingly high risk of rectal and gastric cancer in this family. We identified 12 candidate genes, none was supported as high penetrant, suggesting a complex inheritance. Five of the genes (*DZIP1L, IGSF10, NOTCH1, SF3A1, GAL3ST1*) were more likely than the other seven. The gene best known to be related to cancer was the *NOTCH1*. Further studies are needed to find out more about these variants and other gene variants possibly contributing to the increased cancer risk in this family.
